# Visualization and Experimental Characterization of Wrapping Layer Using Planar Laser-Induced Fluorescence

**DOI:** 10.1021/acsnano.3c07407

**Published:** 2024-01-26

**Authors:** Haobo Xu, Joshua M. Herzog, Yimin Zhou, Yashar Bashirzadeh, Allen Liu, Solomon Adera

**Affiliations:** Department of Mechanical Engineering, University of Michigan, Ann Arbor, Michigan 48105, United States

**Keywords:** planar laser-induced fluorescence (PLIF), slippery liquid-infused porous surfaces (SLIPS), lubricant-impregnated surfaces (LIS), oil-infused surfaces, wrapping layer, wetting ridge, spreading coefficient

## Abstract

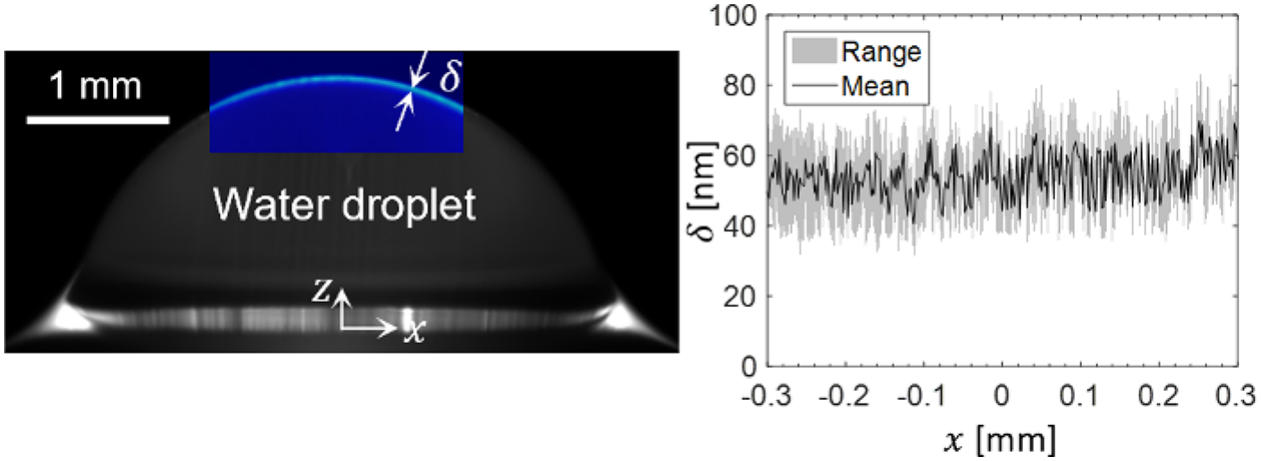

Droplets on nanotextured oil-impregnated surfaces have high mobility due to record-low contact angle hysteresis (∼1–3°), attributed to the absence of solid–liquid contact. Past studies have utilized the ultralow droplet adhesion on these surfaces to improve condensation, reduce hydrodynamic drag, and inhibit biofouling. Despite their promising utility, oil-impregnated surfaces are not fully embraced by industry because of the concern for lubricant depletion, the source of which has not been adequately studied. Here, we use planar laser-induced fluorescence (PLIF) to not only visualize the oil layer encapsulating the droplet (aka wrapping layer) but also measure its thickness since the wrapping layer contributes to lubricant depletion. Our PLIF visualization and experiments show that (a) due to the imbalance of interfacial forces at the three-phase contact line, silicone oil forms a wrapping layer on the outer surface of water droplets, (b) the thickness of the wrapping layer is nonuniform both in space and time, and (c) the time-average thickness of the wrapping layer is ∼50 ± 10 nm, a result that compares favorably with our scaling analysis (∼50 nm), which balances the curvature-induced capillary force with the intermolecular van der Waals forces. Our experiments show that, unlike silicone oil, mineral oil does not form a wrapping layer, an observation that can be exploited to mitigate oil depletion of nanotextured oil-impregnated surfaces. Besides advancing our mechanistic understanding of the wrapping oil layer dynamics, the insights gained from this work can be used to quantify the lubricant depletion rate by pendant droplets in dropwise condensation and water harvesting.

Chemically modified and functionalized surfaces with roughness possess nonwetting properties^1,2^ that are beneficial for a wide range of industrial applications, including antifouling,^3−5^ anti-icing,^6−9^ drag reduction,^10−13^ and phase change.^14−17^ The central theme of nonwettability relies heavily on suppressing contact between the solid substrate and the liquid droplet using an air cushion trapped within the surface texture.^18,19^ State-of-the-art approaches have been based on the “lotus” effect, where intricate nanostructures are carefully designed to maintain an air layer between the structures, forming a stable interface between the substrate and the applied liquid.^20,21^ The pockets of air trapped beneath the droplets placed on these surfaces lead to a composite solid–liquid–air interface in thermodynamic equilibrium. As long as the air pockets within the nanostructures remain stable (for example, by heating the surface above the boiling point),^22^ the surface continues to exhibit superior liquid repellency.

Maintaining stable air pockets, however, is challenging since the trapped air can collapse for a variety of reasons, including mechanical damage (e.g., scratch with a sharp knife), fabrication defects, and external pressure (e.g., droplet impact).^23,24^ The air cushion can also diffuse into the surrounding liquid (especially in underwater applications) and the surface can lose its hydrophobicity.^25,26^ Moreover, lotus-leaf-inspired textured superhydrophobic surfaces^20,21^ are not suitable for organic solvents and low-surface-tension liquids (e.g., hexane ∼18 dyn/cm), since unlike water (∼72 dyn/cm), low-surface-tension fluids can destabilize the air cushion beneath the droplet because of their enhanced ability to completely wet surfaces of even the most hydrophobic materials. Consequently, designing omniphobic surfaces that are capable of repelling a variety of liquids with a wide range of surface tensions is challenging.^2,27^

Early work on omniphobicity employed advanced fabrication techniques to create complex re-entry geometries on surfaces (that is, roughness with overhanging topology) to repel extremely low-energy liquids with surface tensions down to ∼17 dyn/cm (e.g., heptane) at ambient/room temperature and pressure.^2,27,28^ Other approaches to generating reentrant textures (e.g., doubly reentrant structures) that can support strongly metastable composite solid–liquid–air interfaces for excellent omniphobicity were later demonstrated. But still, these strategies of designing super-repellent surfaces rely on air which is not robust (easy to displace), making such hydrophobic surfaces less reliable in practical applications.

A radically different approach to achieving superior nonwetting properties involves replacing the air trapped within the crevices of traditional superhydrophobic surfaces with a more viscous liquid that is more difficult to displace.^29,30^ On these suitably designed nanostructured surfaces infused with a chemically matched lubricant oil (that is, composite hemisolid hemiliquid surfaces), any foreign liquid droplet immiscible with the underlying lubricant will easily slide off at a nearly negligible tilting angle. The inspiration for such surface design is obtained from the *Nepenthes* pitcher plant, which uses a few nanometer thick film of water to inhibit the attachment of insects.^31−33^ These nature-inspired surfaces, which are referred to in current literature as slippery liquid-infused porous surfaces (SLIPS) or lubricant-impregnated surfaces (LIS), are fabricated by replacing the air cushion in conventional nanotextured superhydrophobic surfaces with a chemically matched lubricant oil.^34−37^ By design, these semisolid semiliquid surfaces are “slippery”, a term that refers to the low adhesion of drops on such surfaces as well as the presence of a slip length during droplet motion. Stabilized by the capillary forces that arise from the surface texture, the lubricant used for impregnation (provided it wets the solid substrate preferentially)^38,39^ allows the droplet to move extremely easily, as evidenced by the record-low contact angle hysteresis (<3°).^39−41^ In addition to the low contact angle hysteresis,^42^ textured oil-impregnated surfaces can self-heal by repairing mechanical damages and fabrication defects by reconfiguring/redistributing the lubricant. Oil-infused surfaces have been demonstrated to withstand drop impact pressures of up to 680 atm.^37^ Textured oil-impregnated surfaces are also self-cleaning^43−45^ and omniphobic; that is, they repel a variety of fluids including low-surface-tension liquids (e.g., hexane and other organic liquids) and complex liquids (e.g., blood).^3,46^ Although past studies have demonstrated the potential of lubricant-impregnated surfaces for enhancing various applications, including biofouling prevention,^3,5^ drag reduction,^10,47^ and increased heat transfer rate in condensation^15,17^ and water harvesting,^48,49^ currently these surfaces are not used in real-world applications due to the concern of shear-induced oil depletion.^50−53^

Understanding the oil depletion mechanism and oil depletion rate, which is currently lacking, is the next step in translating oil-impregnated surfaces from benchtop laboratory demonstration prototypes to industrial applications. This work aims to expedite this transition by investigating one of the potential sources of oil depletion: that is, the wrapping oil layer. Besides visually accessing the wrapping layer via a customized planar laser-induced fluorescence (PLIF) imaging setup, the visualization technique developed in this study quantifies the wrapping oil layer thickness with good accuracy (∼tens of nm). The results presented in this study advance our mechanistic understanding of the wrapping layer with the potential to estimate the lubricant depletion rate by pendant droplets during dropwise condensation and water harvesting.

## Results and Discussion

**Figure 1:**
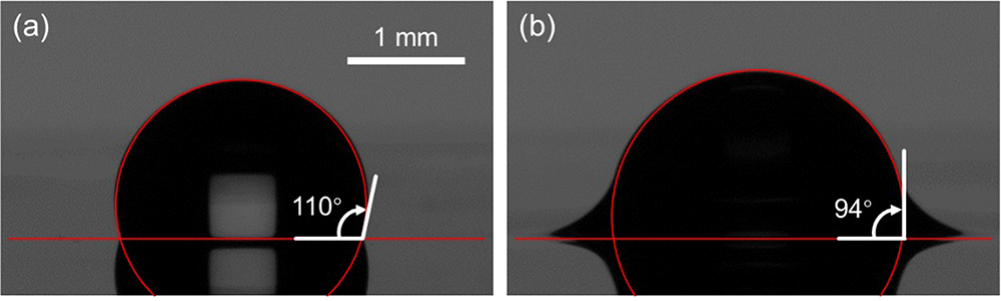
Contact angle. Apparent contact angle of a millimetric water droplet on (a) a Glaco-treated surface and (b) a nanotextured oil-impregnated surface. The apparent contact angle of a water droplet on the Glaco-coated microscope glass slide was ∼110° with an advancing/receding contact angle pair of 113/100° and ∼13° contact angle hysteresis. When the nanotextured glass slide was impregnated with silicone oil, water droplets became highly mobile with an advancing/receding contact angle pair of 95/94° with ∼1° contact angle hysteresis.

We used transparent microscope glass slides as the surface substrates for this study. The glass slides (Fisher Scientific) were functionalized using a nanometer-thick hydrophobic coating of colloidal particles (Glaco Mirror Coat). The fabrication protocol is discussed in detail in Supporting Information S1 with scanning electron microscope images being shown in Figure S1. The droplets used in this study are 5 μL deionized water droplets. The wettability of the Glaco-coated microscope slides was characterized by using a drop shape analyzer (DSA100E, KRÜSS GmbH). The Glaco-coated slides were repellent to water droplets with an apparent contact angle of θ_app_ ≈ 110° as shown in Figure 1a. The advancing/receding contact angle pair of a water droplet on the Glaco-coated surface was 113/100° with 13° contact angle hysteresis.^54,55^ When the surface was impregnated with silicone oil, the apparent contact angle of the water droplets became ∼94° (Figure 1b). The advancing/receding contact angle pair of the nearly hemispherical millimetric water droplet on the nanotextured oil-impregnated surface was 95/94° with a record-low ∼1° contact angle hysteresis. These measurements agree with prior studies that reported ultralow contact angle hysteresis (<3°) of water droplets on textured oil-impregnated surfaces.^37,39^ Replacing the air in lotus-leaf-inspired superhydrophobic surfaces with a more viscous lubricant oil causes the surface to become slippery by minimizing contact line pinning. The measurement uncertainty in contact angle in our experiment was ±3° based on repeated experiments for 1 standard deviation.

From thermodynamic considerations, it is energetically favorable for water droplets to bead up and remain contained without spreading on well-designed textured oil-impregnated surfaces. Additionally, millimetric water droplets on lubricant-infused surfaces have a distinctive feature that is not present on conventional superhydrophobic surfaces. The lubricant oil, that is partly pushed out of the droplet base due to the weight of the droplet,^30^ instantaneously forms an axisymmetric annular wetting ridge near the droplet base because of the imbalance of interfacial forces at the three-phase contact line. The unbalanced vertical component of the surface tension of water pulls the oil, which accumulates at the droplet’s base to form a triangular wedge.^56−58^ Owing to the meniscus shape (concave inward, low-pressure region), lubricant oil continues to be transported to the droplet base from its vicinity.^59^ In this configuration, the boundary between the water droplet and the wetting ridge is not visible to the naked eye.

To visually access the hidden oil–water interface and differentiate between the water droplet and the lubricant, we dyed the silicone oil (Sigma-Aldrich) with Pyrromethene 546 BODIPY dye (C_14_H_17_BF_2_N_2_, Sigma-Aldrich). We used an upright confocal fluorescence laser scanning microscope (LSM 700, Zeiss) to image the droplet and its immediate surroundings. The dye concentration was maintained at 0.1 wt % to reduce its impact on the surface tension of oil and the oil–water interfacial tension. Our interfacial force measurement using the pendant drop method^60,61^ shows that the effect of the dye on the surface tension of oil was insignificant (see Supporting Information S2). The basic premise of this method is that surface tension can be calculated from the shape of a drop as it forms at the end of a capillary tip of a known external radius. The opposing forces of gravity and surface tension determine a droplet’s shape. Thus, one can work backward from a droplet’s shape and the known force of gravity to find its surface tension. This can be done by taking pictures of drop shapes and fitting the classical Laplace equation of capillarity to the droplet’s contours. Even though this equation is a second-order nonlinear equation that does not have an analytical solution, the built-in software of our drop shape analyzer can do this computation for us. For the silicone oil, the oil–air interfacial tension decreased by <1% from 19.81 to 19.75 dyn/cm when fluorescent dye was added at 0.1 wt % concentration (Figure S2a,b).

**Figure 2:**
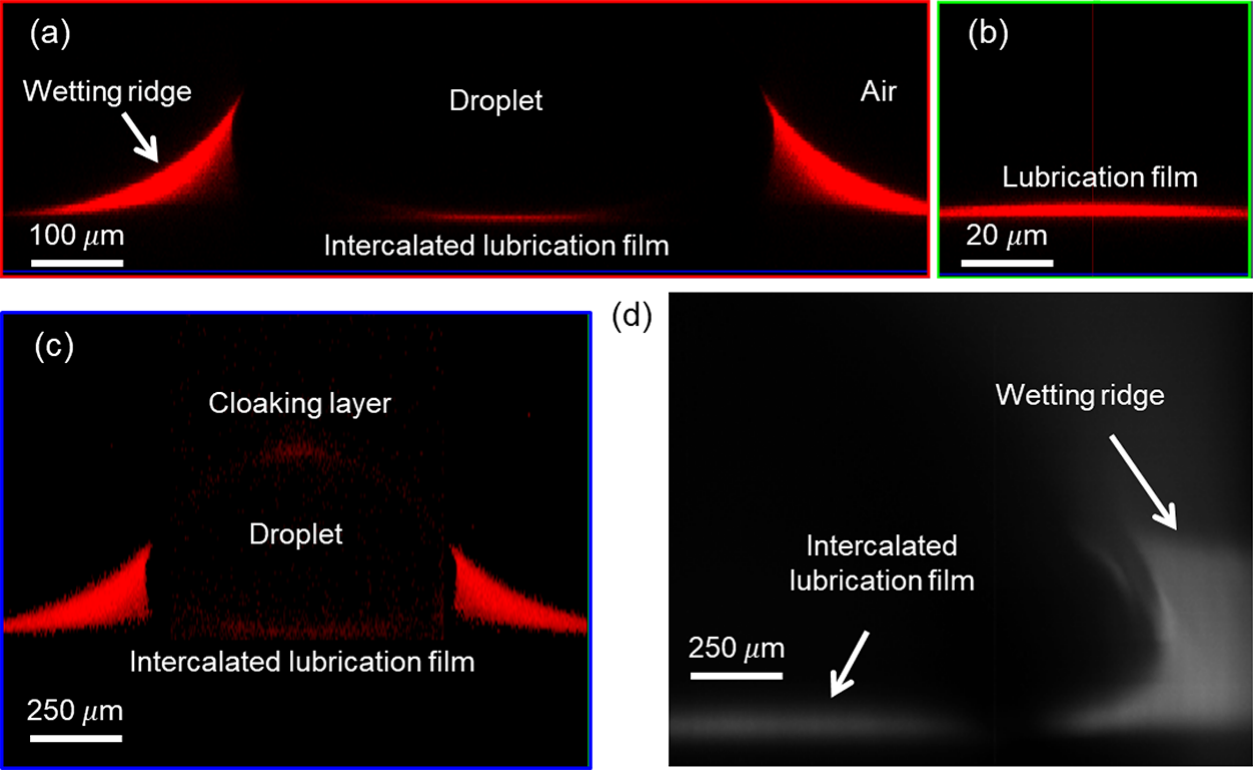
Confocal visualization of the wrapping oil layer. Confocal images of the wetting ridge and wrapping layer when the nanotextured surface was impregnated with (a–c) silicone oil and (d) mineral oil. The silicone oil was dyed using BODIPY (C_14_H_17_BF_2_N_2_) while the mineral oil was dyed using Nile Red (C_20_H_18_N_2_O_2_), both at 0.1 wt % concentration. The wetting ridge and the intercalated oil film became visible when the lubricant oil was labeled by using a fluorescent dye. Unlike mineral oil, silicone oil cloaks the water droplet and forms a wrapping layer.

When the lubricant oil was labeled using BODIPY dye, the contours of the wetting ridge and oil–water interface became visible as shown in Figure 2a–c. The confocal images show that silicone oil cloaks the water droplet and forms a wrapping layer, which agrees with prior studies.^38,56^ In addition to clearly identifying the boundaries of the wetting ridge (oil–air interface) and the otherwise hidden oil–water interface, the confocal images captured in Figure 2a–c show the presence of an intercalated lubricant oil underneath the droplet. This result shows the absence of solid–liquid contact between the applied external liquid (water droplet) and the solid substrate for a well-designed textured oil-impregnated surface. It is the presence of this thin film of oil underneath the droplet that makes water droplets on oil-impregnated surfaces highly mobile by minimizing (and potentially fully eliminating) contact line pinning.^36,38,56^ Moreover, the wrapping layer that encapsulates and cloaks the water droplet is clearly visible in Figure 2c. This cloaking layer is believed to contribute to oil depletion by pendant droplets^51,62^ during dropwise condensation and water harvesting.

In another experiment where we used Nile Red (C_20_H_18_N_2_O_2_, Sigma-Aldrich) to fluorescently label mineral oil (Hydrobrite 380) at 0.1 wt % concentration, we did not detect a wrapping layer as shown in Figure 2d. In this experiment, only the wetting ridge and the intercalated oil film underneath the droplet were visible, while the surrounding air appeared completely dark/black. These results agree with past studies that showed the absence of the wrapping layer when mineral oil was used for impregnation.^50^ The fluorescence images in Figure 2 were captured using a 10× objective on an inverted microscope. A detailed description with a simplified schematic drawing of the confocal experimental setup used for this visualization is provided in Supporting Information S3 and Figure S3.

We corroborated the results shown in Figure 2 (that is, the presence and absence of the wrapping layer for silicone and mineral oils, respectively) by measuring the spreading coefficient of oil on water (*S*_ow_), which is given by , where γ is the interfacial tension between the two phases indicated by the subscripts o, w, and a for oil, water, and air, respectively.^38,56^ The different interfacial tensions were experimentally measured by analyzing the shape of a suspended droplet near its departure by balancing buoyancy and surface tension forces via the pendant drop method.^60^ The results of these measurements are summarized in Table 1. This interfacial force measurement shows that silicone oil has a positive spreading coefficient on water (*S*_ow_ > 0), a result that agrees with our confocal visualization (Figure 2a–c). For water droplets, this is very common since water has a higher surface tension (∼72 dyn/cm) than typical lubricant oils (∼20–30 dyn/cm). One strategy to avoid cloaking is to use a lubricant oil that has a large surface tension. Here we used mineral oil, which has a higher surface tension (∼30 dyn/cm) than silicone oil (∼19 dyn/cm). As shown in Table 1, the spreading coefficient of mineral oil on water is negative (*S*_*ow*_ < 0), again a result that agrees well with our confocal visualization in Figure 2d.

**Table 1:** Spreading Coefficient: Interfacial Tension Measured Using the Pendant Drop Method (All Units in dyn/cm)

oil type	γ_wa_	γ_oa_	γ_ow_	*S*_ow_	wrapping layer?
silicone oil	72.18	18.57	35.99	17.62	yes
mineral oil (Sigma-Aldrich)	72.18	27.87	51.07	–6.76	no
mineral oil (Hydrobrite 380)	72.18	31.47	53.09	–12.38	no
mineral oil (Hydrobrite 550)	72.18	31.50	54.04	–13.36	no

We estimated the thickness of the wrapping oil layer by carrying out an order of magnitude analysis. The competing forces that play a major role in determining the wrapping oil layer thickness are the Laplace pressure and van der Waals (vdW) forces.^63,64^ The scaling for the curvature-induced Laplace pressure (*P*) is *P* ∼ γ*/R*, where γ is the surface tension and *R* is the droplet radius. The opposing disjoining pressure (Π_d_) due to vdW forces, which arises when the oil–air and oil–water interfaces become close to each other within a few nanometers, scales with Π_d_ ∼ *A/*δ^3^, where δ is the separation distance between the two interfaces and *A* is the nonretarded Hamaker constant. Note that for ultrathin liquid films, an additional disjoining pressure term arises due to the intermolecular interactions between the water–oil and oil–air interfaces.^65^ Based on the Lifshitz theory, the nonretarded Hamaker constant for two macroscopic phases (phase 1 and phase 2) interacting across a third medium (phase 3) is given by^65−68^
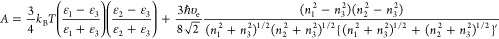
1where *ℏ* is the reduced Planck constant (1.05 × 10^–3^^4^ J s), *n* and ε represent the refractive index and dielectric permittivity, respectively, of the three phases represented by the indices (1, 2, and 3 for water, air, and silicone oil, respectively), *k*_B_ is the Boltzmann constant (1.38 × 10^–2^^3^ J/K), and υ_e_ is the plasma frequency of free-electron gas (5 × 10^15^ s^–^^1^). For a typical water droplet (*R* ≈ 2 mm) encapsulated by a wrapping oil layer, substituting the refractive index and static dielectric permittivity (*n*/ε) for water (1.33/80), air (1.0/1.0), and silicone oil (1.41/2.0) gives a Hamaker constant of *A* ≈ 1.5 × 10^–2^^1^ J. Note that at room temperature, the dominant term in eq 1 is the second term since *k*_B_*T* ≪ *ℏ*υ_e_. Equating the disjoining pressure with the curvature-induced Laplace pressure (*A*/δ^3^ ∼ γ_oa_/*R*) and solving for the wrapping layer thickness (δ) yields
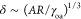
2where γ_oa_ is the surface tension of silicone oil (18.57 dyn/cm, Table 1). This order of magnitude analysis estimates the thickness of the wrapping layer to be δ ≈ 50 nm. Note that the above calculation strongly depends on the Hamaker constant, which is estimated from the refractive index and dielectric permittivity of the three interacting phases (air, water, and silicone oil). More importantly, the Hamaker constant varies based on the separation distance between the two interacting phases.^65^ This, however, is not taken into account in our simplified calculation. Nonetheless, our calculation provides an order-of-magnitude estimate of the thickness of the wrapping layer. We will corroborate this calculation by measuring the wrapping oil layer thickness by using PLIF visualization.

**Figure 3:**
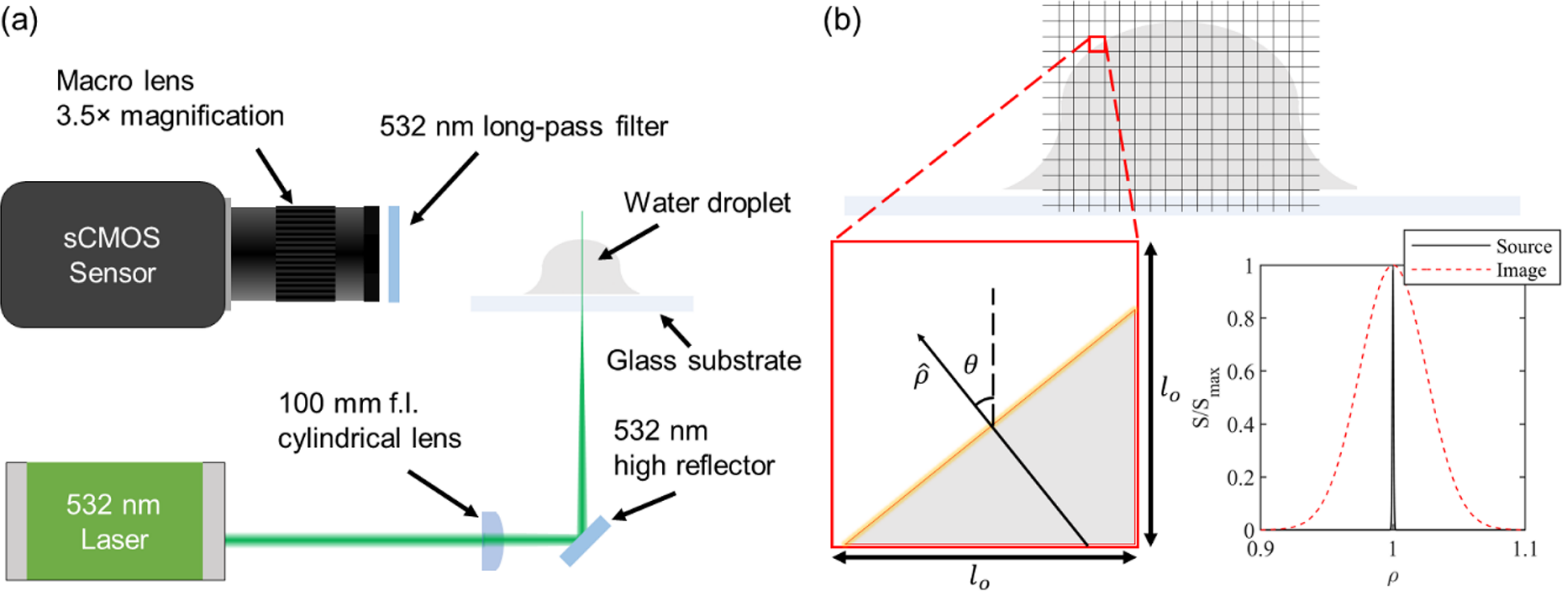
PLIF setup. (a) Optical layout diagram. The experimental setup consists of a laser source, a cylindrical lens, a reflecting mirror, a long-pass filter, and an sCMOS detector. The laser passes through the cylindrical lens, reflects off of a 45° mirror, and passes through the substrate before reaching the water droplet. The emitted light from the dye was allowed to pass through a filter to isolate the emission wavelength from the scattered light. (b) Illustration of droplet geometry, the pixel raster grid, and the line spread function.

The PLIF visualization setup used to measure the wrapping layer thickness consists of five major components: a laser source, a cylindrical lens, a reflector, a long-pass filter, and a scientific complementary metal oxide semiconductor (sCMOS) digital camera with high spatiotemporal resolution, as shown schematically in Figure 3a. The actual experimental setup with a detailed description of the optics is provided in Supporting Information S4 and Figure S4. Briefly, a 532 nm laser beam was transformed into a laser sheet using a cylindrical lens with an anti-reflective coating. The laser sheet was directed to the center of the water droplet. The laser power was maintained at 90 mW to minimize heating and subsequent evaporation of the water droplet, while still providing an adequate signal-to-noise ratio of the fluorescence signal at the photodetector. The sCMOS sensor was outfitted with a variable focal length macro lens and a long-pass filter to reject scattered laser light from the droplet and stray light from the surroundings. Prior to the experiment, the silicone oil was mixed with Nile Red, which has absorption/emission peak wavelengths of 559/635 nm, at 0.1 wt % concentration. The normalized intensity of the fluorescent dye (*S*/*S*_max_, where *S* is the light intensity and *S*_max_ is the maximum light intensity) as a function of the normal vector (, the normalized vector distance between the center of a pixel and the instantaneous center of curvature) (Figure 3b) was computed from the sequence of time-lapse images to identify the total emission intensity for subsequent calculation of the wrapping oil layer thickness.

**Figure 4:**
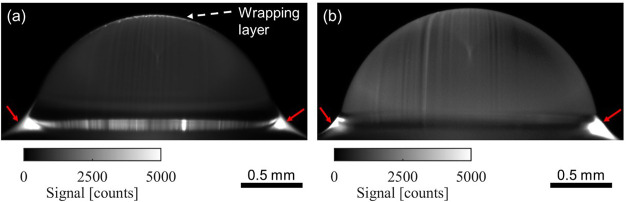
PLIF visualization of the wrapping oil layer. Ensemble-averaged and background-subtracted PLIF image of a water droplet on a transparent glass substrate impregnated with (a) silicone oil and (b) mineral oil. The bright strip on top of the droplet in (a) indicates the presence of a wrapping oil layer. On the other hand, the droplet in (b) does not have a bright strip, indicating the absence of a wrapping layer. The radius of the droplet is 1.0 mm in (a) and 1.1 mm in (b). The radius is measured by circle fitting the droplet spherical cap by a self-developed MATLAB script.

In a typical experiment, we acquired a sequence of high-speed images similar to those shown in Figure 4a. The intensity (*S*) measured on the sensor (in counts, or analog-to-digital units) at a pixel centered at a distance ρ from the oil film along the droplet normal vector is described by the linear PLIF equation as^69^
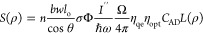
3where *n* is the tracer molecule number density, *b* is the wrapping oil film thickness, *w* is the laser beam waist diameter, *l*_o_ is the pixel length in the object plane, θ is the angle between the oil film normal vector and the pixel axis, σ is the dye absorption coefficient, Φ is the dye fluorescence quantum yield, *I″* is the incident laser fluence, ω is the laser radiation angular frequency, Ω is the lens collection solid angle, η_qe_ is the camera quantum efficiency, η_opt_ is the combined optical efficiency, *C*_AD_ is the analog-to-digital conversion gain, and *L* is the portion of the line-spread function (LSF)^70^ that falls within the pixel boundary. The optical parameters used for the PLIF experiment using silicone oil are given in Supporting Information Table S1. The film thickness (*b*) is calculated using
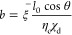
4where  is the integrated normalized intensity from the oil film (calculated as described in the Supporting Information S4), η_c_ is the optical efficiency introduced by the reference cuvette, and χ_d_ is a factor that accounts for dilution of the dye in the reference signal.

Our PLIF visualization experiments show the presence of the wrapping layer in the form of a bright strip at the top of the droplet, as shown by the white dashed arrow in Figure 4a. For this experiment, silicone oil dyed with Nile Red at 0.1 wt % concentration was used for impregnating the Glaco-coated glass slide. On the other hand, when the nanotextured glass slide was impregnated with mineral oil that was labeled using Nile Red at 0.1 wt % concentration, we did not detect a wrapping layer as shown in Figure 4b. This is evident by noting the absence of a white strip (or fluorescent signal) on the outer surface of the water droplet, as shown in Figure 4b. In both of these experiments, a series of 30 images were acquired using the sCMOS camera for analysis. Note that the images shown in Figure 4a,b are ensemble-averaged and background-subtracted fluorescence images used for further quantification of the wrapping layer. Due to the activation of the fluorescent dye molecules, the annular wetting ridge (red solid arrow, Figure 4a,b) is clearly visible irrespective of the oil used for impregnation (silicone or mineral oil).

**Figure 5:**
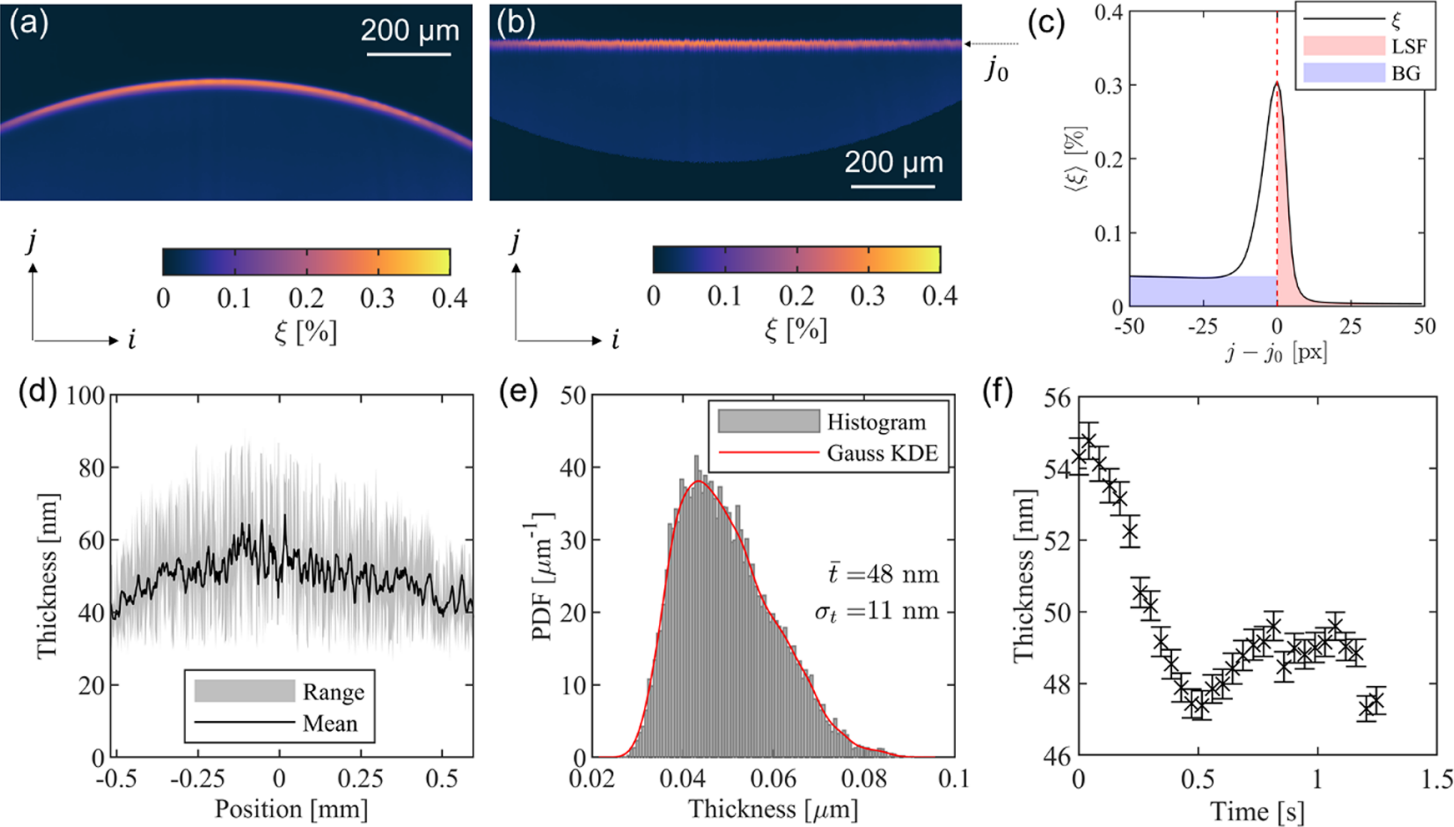
Wrapping layer thickness. (a) PLIF intensity (ξ) image of a water droplet on a silicone oil infused surface. (b) PLIF image shifted to align the wrapping oil film. (c) Corrected intensity profile across the region of interest with the line-spread function (LSF) contribution shaded in red and background (BG) shaded in blue. The total intensity, which is proportional to film thickness, is proportional to the area of the red-shaded region. (d) Mean and range of measured film-thickness profiles calculated from 30 time-lapse images. The horizontal position is the *i* direction in Figure 5b, with the droplet center denoted as the origin *i* = 0. The oil film thickness is nonuniform spatially. (e) Histogram and Gaussian kernel-density estimate (KDE) of film thickness measurements over the entire region of interest. The average oil film thickness is near 48 nm. (f) Temporal variation of the wrapping layer thickness. The error bars represent a 95% confidence interval about the mean. The radius of the droplet was 1.3 mm, and the oil viscosity was 10 cSt.

The fluorescence intensity (ξ) image for the silicone oil, which is corrected for the variation in the laser fluence profile (but not striations introduced by the substrate), is shown in Figure 5a. The location of the wrapping oil layer was then identified in each column of the corrected image as the location of maximum intensity, and each column in the image was shifted such that the wrapping layer appeared at the same pixel coordinate *j* in each column. This is indicated by an arrow and is labeled as *j*_0_ in Figure 5b. The intensity profile along the vertical direction as a function of the distance from the droplet surface averaged over each column is shown in Figure 5c. This analysis clearly shows an intensity peak due to the wrapping layer, the blurring effect of the line-spread function (LSF), and the background (BG) fluorescence visible within the droplet interior. The intensity peak shown in Figure 5c suggests the presence of the wrapping layer for water droplets residing on silicone oil infused textured surfaces. The oil film thickness was calculated using eq 4 after performing the sum as indicated in Figure 5c for each column in each image separately. Furthermore, the film surface orientation in each column (θ) was calculated from the relative vertical offset of the ridge. The ensemble-averaged film thickness profile is shown in Figure 5d with the measured range indicated by the shaded region to account for the variation in our thickness measurement. While there appears to be a significant spatial variation in thickness, it is not immediately clear how much of the observed variation is due to bias caused, for example, by nonuniformity in laser transmission through the substrate. This can easily be caused by surface imperfections due to fabrication defects or dust particles. A histogram of measured oil film thickness values is shown in Figure 5e with a Gaussian kernel-density estimate (KDE) superimposed. The majority of the measurements are centered near 48 nm, although a long tail extends up to 80 nm. The measurement accuracy is taken to be ±50%; a discussion of measurement accuracy is provided in Supporting Information S4.

In addition to the spatial variation, we investigated the temporal variation of the wrapping oil film thickness by analyzing the time-lapse images. This analysis (Figure 5f) shows that the wrapping oil film thickness is nonuniform in time. This result agrees with prior studies that used confocal microscopy and color interferometry to show the nonuniformity of the wrapping layer.^50^ The temporal signal-to-noise ratio in our measurement is expected to be ∼25 at the signal intensities observed in the oil film based on the manufacturer’s specification, which attributes a significant portion of the observed temporal variation to the motion in the oil film. The error bars in Figure 5f represent a 95% confidence interval about the mean from measurements at different positions on a wrapping layer.

Note that the scaling analysis in eq 2 indicates that the wrapping layer thickness does not depend on the initial lubrication film thickness on the surface, which is experimentally validated in Supporting Information S5 and Figures S5–S7.

The oil film thickness (∼50 ± 10 nm) can be used to estimate the rate of oil depletion by falling droplets during dropwise condensation and water harvesting, two practical industrial applications that can benefit from the outcomes of this study. To the first approximation, the oil depletion rate can be calculated by assuming the entire surface area of the droplet (4π*R*^2^, where *R* is the droplet radius) to be covered by oil of thickness ∼50 nm. This first-order approximation, which simply multiplies the surface area of the drop by the wrapping layer thickness, clearly overestimates the depletion rate since droplets on oil-impregnated surfaces are not full spheres but are hemispherical in geometry.

Losing oil by pendant droplets through the wrapping layer is not the only source of oil depletion. The wetting ridge, which is omnipresent, with droplets residing on textured oil-impregnated surfaces, contributes to oil depletion. In fact, the contribution of the wetting ridge to oil depletion can be more significant since its relative size (∼tens/hundreds of micrometers to a few millimeters in radius depending on the initial lubrication film thickness) is significantly larger than the wrapping layer thickness. Note that the wetting ridge is transient in nature since it continues to grow by siphoning oil from the textured surface that accumulates near the droplet base. With the technique developed in this study, that is, by clearly identifying the boundaries of the wetting ridge, the contributions of the wrapping layer and the wetting ridge to lubricant depletion can be quantified.

While our measurement of the wrapping layer thickness agrees well with previous reports, several significant improvements in accuracy can still be made. In particular, the reduction or correction of laser sheet striations by improving substrate quality or implementing improved image processing techniques will greatly reduce uncertainty in the spatial film thickness distribution and mean thickness. Further, the use of a prime lens with a larger, fixed aperture can increase collection efficiency and reduce or eliminate changes in magnification and efficiency between the PLIF and reference images. Additionally, careful cleaning of the substrate can reduce the transmission loss introduced by the surface. Moreover, ensuring repeatable laser alignment and droplet positioning can help reduce the effect caused by different optical conditions. Finally, using an oil film with a known thickness on a flat substrate as a reference image can eliminate errors introduced by optical effects imposed by the cuvette and the bulk dyed oil. Future radiometric and spectroscopic characterization of the dyed oil, especially radiative trapping effects if present, may also lead to significant improvements in the experimental accuracy.

## Conclusions

In summary, we developed a customized planar laser-induced fluorescence optical setup to visualize and measure the thickness of the wrapping oil layer that forms due to the imbalance of interfacial forces at the three-phase contact line when water droplets reside on textured oil-impregnated surfaces. In our work, the wrapping layer was detected by the fluorescence intensity peak that resulted from the absorption of the laser beam by the dye molecules, which are dissolved in the lubricant oil at 0.1 wt % concentration. Our experiments show an intensity peak on the outer surface of the droplet when the oil used for impregnation was silicone oil. This result, which agrees with prior studies, shows the presence of the wrapping oil layer for water droplets deposited on nanotextured silicone-oil-infused surfaces. On the other hand, we did not detect an intensity peak of the fluorescence signal for water droplets residing on nanotextured mineral-oil-infused surfaces, an outcome that suggests the absence of the wrapping layer when mineral oil was used for surface texture impregnation. We corroborated the PLIF results by measuring the spreading coefficient of silicone oil and mineral oil, which involves careful measurement of the different interfacial tensions using the pendant drop method. Consistent with the PLIF visualization results, the pendant drop measurements show that unlike mineral oil, silicone oil forms a wrapping layer, a result that also agrees well with prior studies. More importantly, we measured the wrapping layer thickness using the dye concentration in the lubricant oil as a proxy. This measurement shows ∼50 ± 10 nm wrapping layer thickness, a result that agrees with our order of magnitude analysis (∼50 nm) that balances the curvature-induced Laplace pressure of the oil with the van der Waals forces that arise when the oil–air and oil–water interfaces become a few nanometers apart. The results of this study can be used to estimate the contribution of the wrapping layer to the oil depletion rate in dropwise condensation and water harvesting. The insight gained from this study advances our understanding of the wrapping layer dynamics and its role in lubricant depletion by pendant droplets.

## Methods

### Materials

Silicone oil was supplied by Sigma-Aldrich. Mineral oil was supplied by Sigma-Aldrich and Sonneborn. Nile Red and BODIPY dyes were supplied by Sigma-Aldrich. Methanol was purchased from Sigma-Aldrich. Glaco spray coating was supplied by SOFT99. All chemicals were used as is without further purification. Glass slides were purchased from Fisher Scientific.

### Sample Fabrication

The microscope glass slides were wet cleaned, dried using compressed nitrogen gas, and plasma treated for 15 min. This was followed by immersing and slowly pulling out the glass slide vertically from the Glaco–methanol solution. Finally, the Glaco-coated glass was dried by placing it inside a convection oven at 70 °C for 2 h. The glass slides were impregnated with lubricant oil for the experiment. The excess oil was removed by gravity by positioning the samples for >12 h vertically.

### Contact Angle Measurement

A drop shape analyzer (DSA100E, KRÜSS GmbH) was used to measure the static/equilibrium and advancing and receding contact angles of a water droplet residing on a glass slide coated with silicone oil. The static contact angle of a sessile water droplet was measured by analyzing the shape of a 5 μL sessile droplet using the built-in software of the drop shape analyzer. The advancing and receding contact angles were measured by adding and withdrawing 5 μL of water on a 10 μL sessile droplet. Water was added and withdrawn slowly at 0.05 μL/s to avoid dynamic effects. When water was added to the sessile droplet, the contact line advanced and, hence, the advancing contact angle. Similarly, when water was withdrawn from the sessile droplet, the contact line receded and, hence, the receding contact angle. The advancing and receding contact angles were measured by analyzing the advancing and receding contact lines that were captured at 10 fps. The contact angle hysteresis was calculated by taking the difference between the advancing and receding contact angles.

## Supporting Information Available

Sample fabrication, interfacial force measurement, confocal imaging, PLIF measurement, and wrapping layer dynamics (PDF)
