# Supplementary material for: Visualization and Experimental Characterization of Wrapping Layer Using Planar Laser-Induced Fluorescence
Source: ACS Nano. 2024 Jan 26;18(5):4068–76. doi: 10.1021/acsnano.3c07407 (PMC10851937; doi:10.1021/acsnano.3c07407)
Supplement: Supplementary file 1 — nn3c07407_si_001.pdf [file nn3c07407_si_001.pdf]

Supporting Information for
Visualization and experimental characterization of wrapping layer using planar laser-induced fluorescence

Haobo Xu[1], Joshua M. Herzog[1], Yimin Zhou[1], Yashar Bashirzadeh[1], Allen Liu[1], Solomon Adera[1,]*
[1]*Department of Mechanical Engineering, University of Michigan, Ann Arbor, MI 48105, USA*

*Corresponding author: Solomon Adera
Email: sadera@umich.edu

S1. SAMPLE FABRICATION

The test samples for this study were fabricated from a 1.0 mm thick 3in.×1in. microscope fused glass slides (12-550-123, Fisher Scientific). The glass slides were immersed in an Alconox solution (1404-1 Detergent, Alcojet) and sonicated for 20 min (CPX-1510, Branson Ultrasonics). Following sonication, the samples were wet cleaned using acetone, methanol, and isopropyl alcohol. After wet cleaning, the samples were rinsed thoroughly using de-ionized water and dried using compressed nitrogen gas. This was followed by a 15-minute plasma treatment (PDC-001-HP, Harrick Plasma) in a nitrogen environment. The clean and plasma-treated glass slides were hydrophobized by coating them with colloidal particles of nanometric thickness (Glaco Mirror Coat, Soft 99). To cover the glass slides uniformly with a hydrophobic coating, the Glaco solution was first mixed with methanol to form a dilute solution. After fully immersing the glass slides into the Glaco-methanol solution, the samples were pulled out of the solution vertically at a controlled speed. Finally, the samples were placed in a forced air convection oven (VWR 1350 FM) that was maintained at 240 °C for 2 hours. We characterized the surface topography of the Glaco-coated glass slides using scanning electron microscopy (Tescan Mira3 FEG-SEM). The SEM images in Fig. S1 show a nearly uniform Glaco coating.

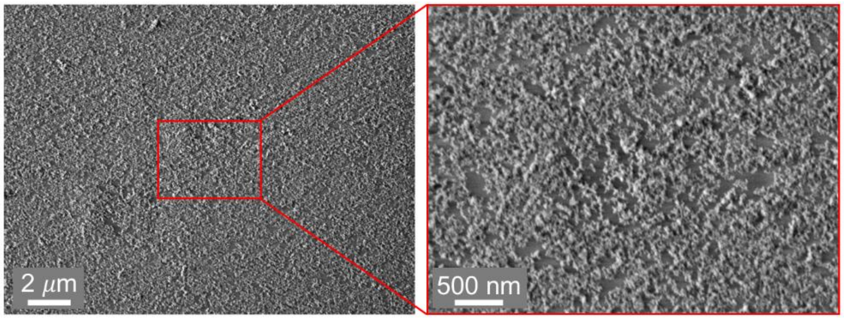

Figure S1. Surface characterization. Scanning electron microscopy (SEM) images of the microscope glass slide after surface treatment. The glass slide was chemically treated and coated with hydrophobic colloidal particles. The hydrophobic coating, which is nearly uniform as can be seen from the magnified inset image, is few nanometers thick. The SEM images are taken at a 10° tilting angle.

S2. INTERFACIAL FORCE

The lubricant oil used to characterize the wrapping oil thickness (that is, silicone oil) was fluorescently labelled using Nile Red (72485-100MG, Sigma-Aldrich) at 0.1 wt% concentration. The surface tension of silicone oil was measured before and after dissolving the fluorescent dye in the lubricant oil. The interfacial tension (IFT) was measured by analyzing the shape of a suspended droplet near departure. A typical millimeter-sized pendant drop suspended from a stainless-steel needle is shown in Fig. S2a.

During interfacial force measurement, the silicone oil was pushed out from the needle slowly at 0.1 μl/min to minimize the effect of fluid motion on the droplet shape. High-resolution images (1200×1200 pixels at 150 fps on an 11×7 mm sensor) of the pendant drop near departure were captured at 10 fps and analyzed using a drop shape analyzer (DSA100, KRÜSS GmbH). The calculated IFT (oil-air surface tension) for pure silicone oil without the dye (0 wt%) was 19.81±0.07 dyn/cm. However, when Nile Red ($C_{20}H_{18}N_2O_2$)

was dissolved in the oil (0.1 wt%), the surface tension decreased to 19.75±0.10 dyn/cm. The error bars were obtained from repeated experiments with one standard deviation. The resolution of the drop shape analyzer is 0.01 dyn/cm. The change in surface tension of silicone oil due to the addition of Nile Red is <1% and is shown in Fig. S2b.

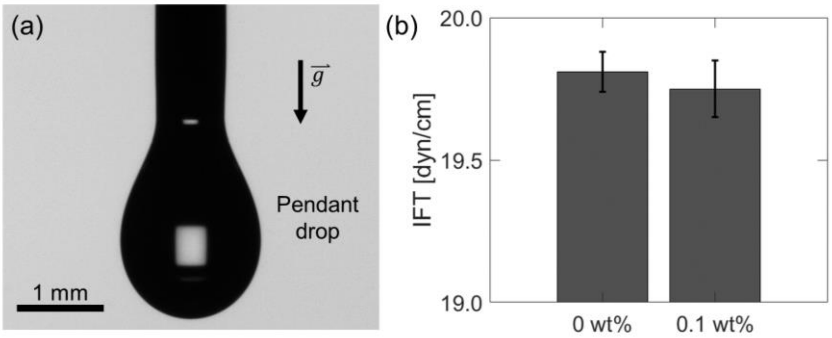

Figure S2. Interfacial tension measurement. (a) Photograph of a typical pendant drop suspended from a stainless-steel needle. A drop shape analyzer was used to calculate the interfacial tension (IFT) from the droplet shape. (b) IFT (oil-air surface tension) of pure (0 wt%) and dyed (0.1 wt%) silicone oil. The surface tension of silicone oil decreased by <1% (from 19.81 to 19.75 dyn/cm) when Nile Red was added at 0.1 wt% concentration.

S3. CONFOCAL IMAGING EXPERIMENTAL SETUP

Confocal images were taken using an oil immersion Plan-Apochromat 10×/1.4 NA objective on an inverted microscope (Olympus IX-81) equipped with an iXON3 electron multiplier charge-coupled device (EM-CCD) camera (Andor Technology), an OBIS LS/LX laser (Coherent), and a USB 6003 data acquisition device (National Instruments) for controlling lasers (Fig. S3). In this experiment, a Yokogawa CSU-X1 spinning disk confocal was used for acquiring time-lapse images. Image acquisition was controlled by MetaMorph (Molecular Devices). The vertical z-stack images were captured with 488 nm excitation at an exposure time of 500 ms. The 3D z-projected images were produced by the brightest point projection of z-stack image sequences using Fiji/ImageJ.

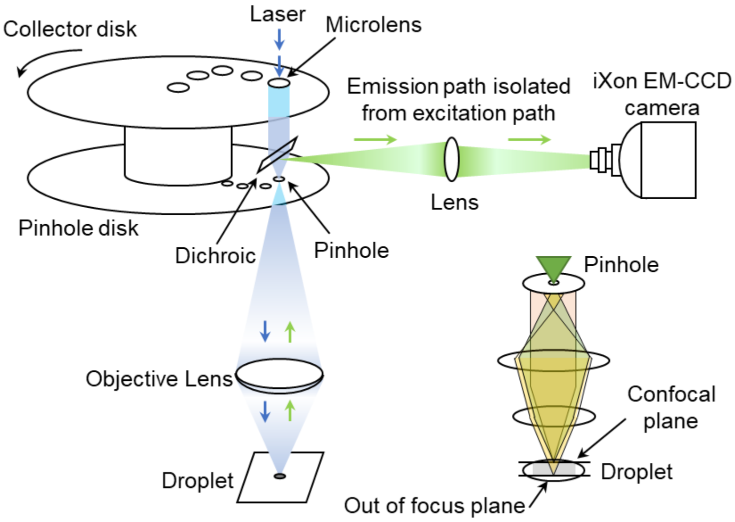

Figure S3. Confocal imaging setup. A pattern of microlenses on a collector disk focuses a filtered laser beam on an array of spinning disk pinholes. By passing through the pinholes and an objective lens, the exciting light illuminates the specimen at the confocal plane. Here, the specimen is a water droplet residing on a transparent microscope glass slide impregnated with oil. Prior to the experiment, the lubricant oil was fluorescently labelled using Nile Red at 0.1 wt% concentration. The light emitted by the excited dye molecules follows the same path as the exciting light except they get reflected from the dichroic mirror, filtered, and focused onto the highly sensitive detector camera using a lens. The confocal plane was varied stepwise in the vertical direction to acquire z-stack 2D fluorescent images of the water droplet.

S4. PLANAR LASER-INDUCED FLUORESCENCE (PLIF) SETUP

We developed a custom-made planar laser-induced fluorescence (PLIF) optical setup. The experimental setup consists of five main components (Fig. S4): a laser source, a cylindrical lens, a 45° reflector, a long-pass filter (>532 nm), and a scientific complementary metal-oxide semiconductor (sCMOS) sensor. The laser source (OBIS LX 532 100 mW, Coherent) was maintained at room temperature by attaching it to a convectively cooled heat sink (1193289, Coherent). The 532 nm laser beam was transformed into a laser sheet using an anti-reflection coated cylindrical lens (37-594, Edmund) and directed onto the center of the droplet with a 532 nm high-reflector 45° mirror (NB1-K12, ThorLabs). The laser sheet was allowed to pass through the droplet from beneath, where the optical interface is flat, to avoid distortions and beam steering. The beam waist diameter was estimated to be 100 μm at the focal point in the oil film based on Gaussian beam optics using the manufacturer-specified beam diameter of 0.7 mm and M^2 value of 1.1. This value also corresponds to a Rayleigh range (depth of field) of 15 mm, significantly larger than the region of interest. The laser was operated at 90 mW to maximize fluorescence signal-to-noise ratio, corresponding to an average irradiance of 130 MW/cm^2. The sCMOS sensor (ORCA-Fusion C14440-20UP, Hamamatsu) was outfitted with a variable focal length macro photo lens (MP-E 65mm f/2.8 1-5× Macro Photo, Canon), adjusted to approximately 3.5 times magnification (f-number≈12.6). As per the manufacturer's specification sheet by Canon, the effective f-number for 3× and 4× magnifications are 11.2 and 14.0, respectively. We took the average f-number for 3.5× magnification. This arrangement of the optics corresponds to an object-plane pixel length of 1.835 μm, approximately 10 μm imaging resolution based on the Rayleigh Criterion, and a depth of field of approximately 0.4 mm. A 532 nm long-pass filter (BLP01-532R-25, Semrock) was used to reject scattered laser light. The sCMOS was exposed for 5 ms for oil film measurements and operated in ultra-quiet scan mode to minimize thermal noise. The oil used for impregnation was dyed with Nile Red ($C_{20}H_{20}N_2O_2$, Sigma-Aldrich), which is soluble in silicone oil but not in water. The dye concentration was kept at 0.1 wt% to preserve the thermophysical property of the lubricant oil, particularly the surface tension.

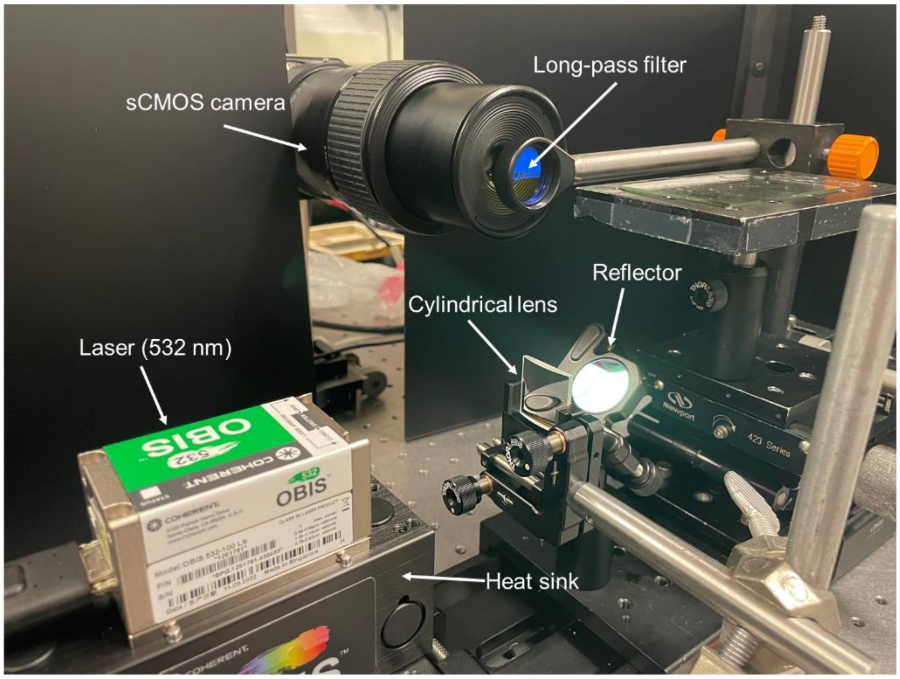

Figure S4. PLIF experiment setup. The PLIF setup consists of five components: a laser source, a cylindrical lens, a reflecting mirror, a long pass filter, and a sCMOS camera. The laser source, which is attached to a heat sink to suppress overheating, generates laser at 532 nm wavelength. The laser passes through the cylindrical lens, reflects off of a 45° mirror, and passes through a transparent substrate before reaching the water droplet. The laser excites the dye molecules in the lubricant oil. The emitted light from the dyed oil is allowed to pass through a long-pass filter to isolate the light emitted by the oil (fluorescent molecules) from the surrounding light.

In a typical experiment, we acquire a series of time-lapse images. The intensity measured on the sensor (in counts, or analog-to-digital units) at a pixel centered at a distance ρ from the oil film along the droplet normal vector is described by the linear PLIF equation

$$S(\rho) = n\frac{bwl_o}{\cos\theta}\,\sigma\Phi\frac{I''}{\hbar\omega}\frac{\Omega}{4\pi}\,\eta_{qe}\,\eta_{opt}\,C_{AD}\,L(\rho), \tag{1}$$

where n is the tracer molecule (Nile Red) number density in the oil, b is the oil film thickness, w is the laser beam waist diameter, l_o is the pixel length in the object plane, θ is the angle between the oil film normal vector and the pixel axis (such that $-\pi/4 \leq \theta \leq \pi/4$; refer to Fig. 3b), σ is the dye absorption cross-section, Φ is the dye fluorescence quantum yield, I'' is the incident laser fluence, $\hbar$ is the reduced Planck's constant, ω is the laser radiation angular frequency, Ω is the lens collection solid angle, η_{qe} is the camera quantum efficiency, η_{opt} is the combined optical efficiency, and C_{AD} is the analog-to-digital conversion gain. Finally, the factor $L(\rho)$ represents the portion of the line-spread function (LSF) that falls within the pixel boundary. The LSF describes the blurring caused by diffraction (or optical aberrations for imperfect lenses) when imaging a one-dimensional object; a detailed discussion of the LSF and related parameters, and their effect on imaging can be found in a prior study.[1] The LSF width is on the order of the imaging resolution, which is approximately 10 μm or 5-6 pixels. Since the oil film thickness is expected to be significantly smaller than the LSF width, the LSF must be accounted for explicitly.

Many of the parameters in the PLIF equation are not known accurately and may vary slowly within an image or between measurements. To account for this variation, a measurement of a cuvette containing dyed oil is taken as a reference. The PLIF equation for the dyed oil in the cuvette is similar to Eq. (1),

$$S_{ref}(\rho) = S_{ref} = nwl_o^2\,\sigma\Phi\,I''\frac{\Omega}{4\pi}\,\eta_{qe}\,\eta_{opt}\,\eta_c\,\chi_d\,C_{AD}, \tag{2}$$

where S_{ref} is the signal measured from the reference cell, χ_d is a factor to account for dilution (if necessary) of dye in the reference experiment, and η_c is the transmission and other optical efficiencies introduced by the cuvette (e.g., cuvette wall transmission). Unlike Eq. (1), the reference measurement does not depend explicitly on ρ because the intensity distribution in the object plane is locally uniform; the light that is diverted from a pixel due to optical aberrations is compensated by adjacent pixel volumes in the object plane. The normalized intensity from the oil film, ξ, is then given by,

$$\xi(\rho) = \frac{S(\rho)}{S_{ref}} = \frac{b}{l_0\cos\theta}\,\eta_c\,\chi_d\,L(\rho). \tag{3}$$

Since the LSF is not known in general, a summation is performed along ρ at each location on the droplet surface. Summing over ξ yields,

$$\sum_j \xi(\rho_j) = \bar{\xi} = \frac{b}{l_0\cos\theta}\,\eta_c\,\chi_d, \tag{4}$$

where the sum is performed over all pixels j along the droplet normal vector. This removes the dependence on the LSF since by definition $\sum_j L\left(\rho_j\right) = 1$. In general, this procedure may require careful interpolation and resampling of the image. Here, for simplicity and due to the relatively small curvature of the droplet in the region of interest, the sum is performed over the vertical pixel direction.

Although luminescence and Raman scattering from the droplet and air surrounding the oil film are generally negligible, some background fluorescence is visible within the droplet image caused by the fluorescence of oil beneath the droplet. The droplet acts as a lens and redirects the fluorescence emission toward the camera, causing a modest background that appears to originate from the droplet interior. To avoid bias from this effect, the direct summation over pixel intensities is replaced by,

$$\bar{\xi} = \xi(\rho_{j_0}) + 2\sum_{j>j_0}\xi(\rho_j), \tag{5}$$

where j_0 is the pixel index corresponding to the peak intensity in the oil film, and the sum is taken over pixels that lie above the droplet boundary along the droplet surface normal vector. The factor of two is

included to account for the portion of the LSF that is obscured by the background luminescence, exploiting the symmetry of the LSF. Finally, the film thickness (b) is calculated using

$$b = \bar{\xi}\,\frac{l_0 \cos\theta}{\eta_c\,\chi_d}. \qquad (6)$$

The technique was performed using silicone oil, where a wrapping oil layer is expected to form around the droplet. Mineral oil where no wrapping oil layer is expected was also used in our experiments. For each measurement, a series of 30 time-lapse images were acquired. The ensemble-averaged background-subtracted fluorescence intensity (S) images for silicone and mineral oil are shown in Fig. 4a and 4b, respectively. Comparing the figures in Fig. 4a-b, the water droplet residing on a silicone oil-infused glass substrate exhibits a bright strip at its top (white dashed arrow, Fig. 4a), indicating the presence of the wrapping oil layer. On the other hand, the droplet in Fig. 4b is dark without the bright strip, indicating the absence of the wrapping oil layer. Estimated optical parameters for the PLIF experiment using silicone oil are listed in Table S1. From Table S1, the dominant uncertainty is the lens collection fraction which is caused by uncertainty in magnification and f-number. For a perfect optical setup, the relative thickness measurement made here is independent of this value because it appears in both the PLIF image intensity and reference image intensity. However, due to uncertainty in the positioning of the cuvette for the reference image, this factor is not perfectly cancelled out. The relative uncertainty in the lens collection fraction thus serves as a conservative upper limit on bias with a magnitude of ≈33%. A lower bias limit can be estimated from the remaining parameters that are not explicitly cancelled out in the analysis (l_o, η_c, χ_d) with a magnitude of ≈3% assuming the individual uncertainties are uncorrelated. Finally, additional sources of error not explicitly estimated here include beam distortions introduced by the substrate or droplet, temporal variation in laser irradiance, concentration dependence of dye fluorescence, and fluorescence reabsorption. Laser sheet striations appear in the PLIF image data in the oil layer beneath the droplet with intensity variations on the order of 40% (standard deviation) of the mean. A similar variation in fluorescence intensity per molecule due to quenching or reabsorption was observed within the range of dilutions used for the reference image. It is not clear precisely how these observations relate to the measurement bias so, for simplicity, the bias magnitude is conservatively taken to be 50%.

Table S1. Estimated optical imaging parameters for PLIF experiment. Values are measured or calculated directly except σ and Φ, which are properties of the fluorescent dye (Nile Red) in Dimethyl Sulfoxide (DMSO).[2]

Symbol	Description	Value	Uncertainty	Unit
l_0	Object-plane pixel length	1.835	±0.02	μm
η_c	Cuvette transmission efficiency	0.97	±0.01	-
χ_d	Reference oil dilution factor	400	±10	-
n	Dye number density	1.1×10^{16}	$\pm1.0\times10^{15}$	mm^{-3}
w	Laser beam waist diameter	100	±10	μm
σ	Dye absorption cross-section	6.0×10^{-17}	-	cm^2
Φ	Dye fluorescence quantum yield	0.4	-	-
f	Lens f-number	12.6	±1.4	-
$\Omega/4\pi$	Lens collection fraction	2.4×10^{-4}	$\pm8.0\times10^{-4}$	-
I''	Incident laser fluence	6500	±100	J/mm^2
$\hbar\omega$	Photon energy	3.7×10^{-19}	$\pm0.1\times10^{-19}$	J
η_{qe}	Camera quantum efficiency	0.75	±0.05	e$^-$/photon
η_{opt}	System optical efficiency	0.90	±0.05	-
C_{AD}	Analog-to-digital conversion gain	0.23	±0.01	e$^-$/ADU

S5. EFFECT OF INITIAL LUBRICANT THICKNESS ON WRAPPING LAYER

The dynamics of the wrapping oil layer that encapsulates the outer surface of the droplet is different from the wetting ridge, which partially blocks the droplet base by forming an oil skirt. Our scaling analysis shows that the wrapping layer thickness does not depend on the initial lubrication film thickness or the wetting ride height. Neither does it depend on the surface micro/nanotexturing of the surface and lubricant viscosity.

To validate this claim, we conducted experiments where we measured the wrapping oil layer thickness by varying the initial lubricant film thickness from 6 μm to 12 μm. This result, which is shown in Figure S5a-b, shows that the wrapping layer thickness is nearly identical (≈50 nm) for thick (12 μm) and thin (6 μm) initial lubricant film thickness when the lubricant viscosity was maintained constant at 10 cSt. The initial lubricant film thickness was measured via white light interference microscopy using a spectrometer (USB2000+ Fiber Optic SpectraSuite, Ocean Optics).

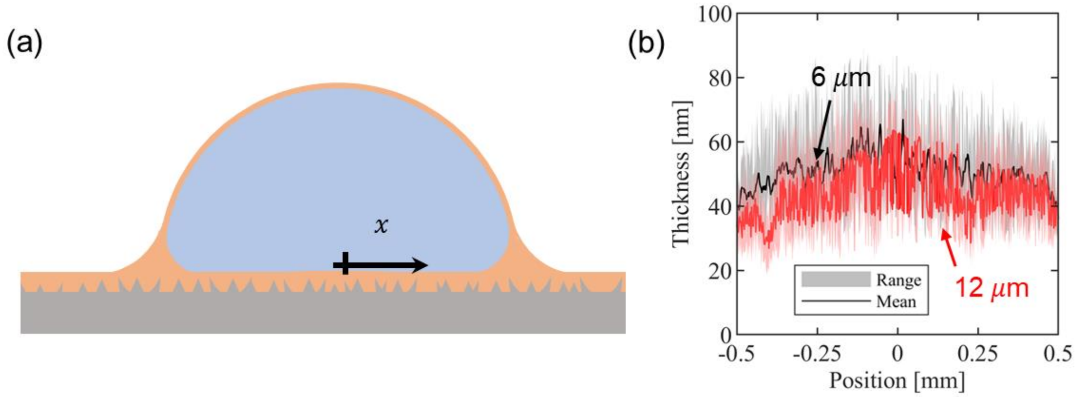

Figure S5: Wrapping layer thickness measurement. (a) Schematic of a droplet on a textured oil-impregnate surface. (b) Spatial variation of the wrapping oil layer thickness. In our experiments, the wrapping oil layer thickness remained nearly the same at 50 nm when the initial lubricant film thickness was doubled from 6 μm to 12 μm while keeping the lubricant viscosity constant at 10 cSt.

The growth rate of the wetting ridge and the corresponding ridge height (h_{wr}, Figure S6a) depends on the initial thickness of the lubrication film (h_{lub}, Figure S6a). To clarify this point, we conducted experiments where we deposited a 10 μl water droplet on an oil-infused silicon micropillar structure. For these experiments, the silicon micropillars, which were fabricated using contact photolithography and deep reactive-ion etching,[3-5] have diameter, height, and center-to-center spacing of ≈10 μm, ≈20 μm, and ≈15 μm, respectively. After silanization, the micropillars were impregnated with silicone oil of varying viscosity (5-500 cSt). Furthermore, the thickness of the lubrication film, which was measured using white light interference microscopy, was varied between 5 μm and 20 μm. The data for the 6 μm and 12 μm lubrication film thickness (viscosity = 10 cSt) is shown in Figure S6b-c. We captured images of the wetting ridge at 30 frames per second and analyzed the images using MATLAB and ImageJ. The results of these experiments show that the wetting ridge growth rate (both wetting ridge height h_{wr} and wetting ridge volume V_{wr}) is faster when the initial lubrication film thickness is 12 μm (compared to the 6 μm thickness) as shown in Figure S6b-c. These results show that the height of the wetting ridge (h_{wr}) increases with the initial lubrication film thickness, a result that agrees with prior studies.[6, 7] The wetting ridge volume in Figure S6c is calculated by analyzing the time-lapse images using MATLAB as discussed in detail in our recent publication.[8, 9]

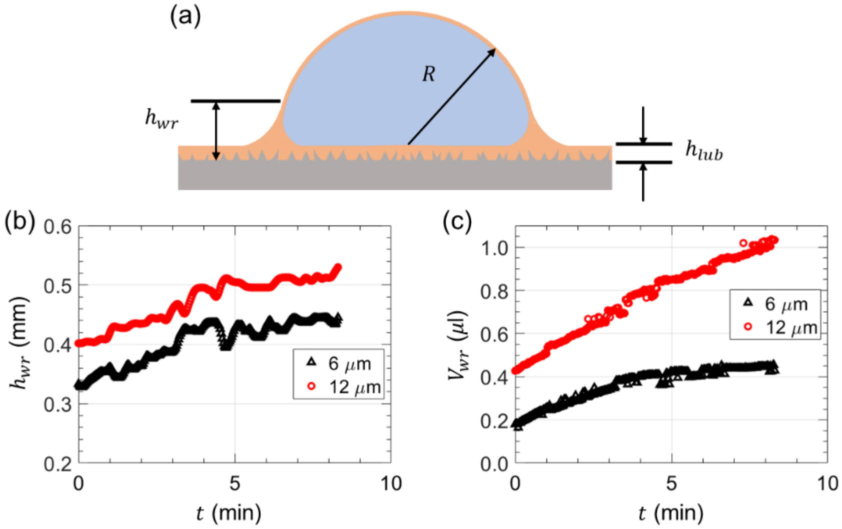

Figure S6: Wetting ridge growth rate. (a) Schematic of a 10 μl water droplet residing on an oil-impregnated silicon micropillar structure showing the wetting ridge height (h_{wr}) and lubricant film thickness (h_{lub}). Temporal variation of the wetting ridge height (b) and wetting ridge volume (c). The growth of the wetting ridge height (h_{wr}) and wetting ridge volume (V_{wr}) increases with initial lubrication film thickness. Compared to 6 μm lubricant film thickness, the 12 μm lubricant thickness gives rise to larger wetting ridge height h_{wr} and wetting ridge volume V_{wr}.

We believe that wetting ridge growth dynamics is determined by the balance of interfacial forces at the three-phase contact line near the droplet base. A water droplet was deposited on a lubricant-infused surface and time-lapse images were captured using the built-in camera in our drop shape analyzer (DSA100E, KRÜSS GmbH). The result shows that the wetting ridge radius grows at a faster rate initially and slows down as it approaches the droplet radius (Figure S7a-b). This result suggests the presence of a steady-state growth rate of the wetting ridge, which may depend on the droplet volume, initial lubricant film thickness, lubricant viscosity, and the micro/nanostructuring of the underlying surface. This experimental result can be rationalized by looking at the Young-Laplace equation, which gives the wetting ridge pressure (P_{wr}) as $P_{wr} = P_{amb} + \gamma_{oa}(1/r_1 + 1/r_2)$, where P_{amb} is the ambient pressure, and r_1 and r_2 are the two principal radii of the oil menisci (Figure S7a), and γ_{oa} is the surface tension of the lubricant oil. Steady-state wetting ridge shape suggests that the two radii of curvatures must be equal in magnitude and opposite in direction (that is, $|r_1| = |-r_2|$) since $1/r_1 + 1/r_2 = 0$. Note that the positive and negative signs on the meniscus radii indicate inward and outward curvatures, respectively. This analysis supports the result in Figure S7b, which shows asymptotically approaching wetting ridge radius (r_1) and droplet base radius (r_2).

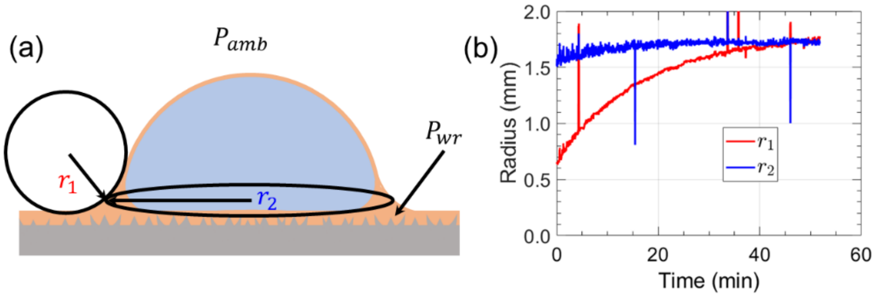

Figure S7: Steady-state growth rate of the wetting ridge. (a) Schematic of the wetting ridge, which emerges when a droplet is deposited on a lubricated surface due to the imbalance of interfacial forces at the contact line. (b) Wetting ridge (r_1) and droplet base radius (r_2) as a function of time. The wetting ridge radius asymptotically approaches the droplet base radius, suggesting the presence of a steady-state wetting ridge growth rate, shape, and volume. At steady state, r_1 and r_2 are equal in magnitude and opposite in direction ($|r_1| = |-r_2|$) since $1/r_1 + 1/r_2 = 0$ to satisfy the equilibrium condition for the Young-Laplace equation (that is, zero pressure difference between the droplet and its surrounding).

REFERENCES

1. Hornak, J. P., *Encyclopedia of Imaging Science and Technology*; Wiley: 2002; pp 390-419.
2. Martinez, V.; Henary, M., Nile red and nile blue: applications and syntheses of structural analogues. *Chem. Eur. J.* **2016,** *22* (39), 13764-13782.
3. Adera, S.; Antao, D.; Raj, R.; Wang, E. N., Design of micropillar wicks for thin-film evaporation. *Int. J. Heat Mass Transfer* **2016,** *101*, 280-294.
4. Raj, R.; Adera, S.; Enrigh, R.; Wang, E. N., Polygonal droplets on microstructured surfaces. *J. Heat Transfer* **2014,** *136* (8).
5. Raj, R.; Adera, S.; Enright, R.; Wang, E. N., High-resolution liquid patterns via three-dimensional droplet shape control. *Nat. Commun.* **2014,** *5* (1), 1-8.
6. Sahoo, S.; Mukherjee, R., Evaporative drying of a water droplet on liquid infused sticky surfaces. *Colloids Surf. A Physicochem. Eng. Asp.* **2023,** *657*, 130514.
7. Üçüncüoğlu, R.; Erbil, H. Y., Water Drop Evaporation on Slippery Liquid-Infused Porous Surfaces (SLIPS): Effect of Lubricant Thickness, Viscosity, Ridge Height, and Pattern Geometry. *Langmuir* **2023,** *39* (18), 6514-6528.
8. Xu, H.; Zhou, Y.; Daniel, D.; Herzog, J.; Wang, X.; Sick, V.; Adera, S., Droplet attraction and coalescence mechanism on textured oil-impregnated surfaces. *Nat. Commun.* **2023,** *14* (1), 4901.
9. Gandee, H.; Zhou, Y.; Lee, J.; Chomali, J.; Xu, H.; Adera, S., Unique ice dendrite morphology on state-of-the-art oil-impregnated surfaces. *Proc. Natl. Acad. Sci. USA* **2023,** *120* (1), e2214143120.
